# Risk factors and lethality associated with Candidemia in severe COVID-19 patients

**DOI:** 10.18502/cmm.8.1.9212

**Published:** 2022-03

**Authors:** Zehra Beştepe Dursun, Hilal Sipahioğlu, Recep Civan Yüksel, Hafize Sav, İlhami Çelik

**Affiliations:** 1 Health Science University, Kayseri Faculty of Medicine, Department of Infectious Diseases, Kayseri, Turkey; 2 Health Science University, Kayseri Faculty of Medicine, Department of Intensive Care Unit, Kayseri, Turkey; 3 Health Science University, Kayseri Faculty of Medicine, Department of Mycology, Kayseri, Turkey

**Keywords:** COVID-19, Candidemia, Intensive care unit, Risk factor

## Abstract

**Background and Purpose:**

Candidemia remained important in the intensive care units (ICU) during the COVID-19 pandemic. This study aimed to investigate the clinical and laboratory data on candidemia in COVID-19 patients.

**Materials and Methods:**

The baseline characteristics, as well as laboratory and clinical findings of candidemia and non-candidemia patients, were compared. Candidemia was defined as the isolation of Candida spp. from blood cultures. The isolates were identified by VITEK® 2 (bioMérieux, France) commercial method. Antifungal susceptibility was assessed using the E-test method. Univariate and multiple binary logistic regression analyses were performed to compare the variables.

**Results:**

In total, 126 patients with the COVID-19 disease were included. Candidemia was diagnosed in 44 (35%) of the patients. The number of patients with diabetes mellitus and chronic renal failure was higher in the candidemia group. In the candidemia group, the duration of ICU stay of patients, the 30-day mortality rate, mechanical ventilation therapy, and systemic corticosteroids (Prednisone) usage were significantly higher in candidemia patients. Moreover, the median white blood cell, neutrophils, and lactate dehydrogenase were higher in the candidemia group.

Univariate and multiple binary logistic regression analyses were performed to compare the variables. Isolated species were identified as *Candida albicans* (n=12, 41%), *Candida parapsilosis* (n=7, 24%), *Candida glabrata* (n=6, 21%), *Candida tropicalis* (n=3, 10%), and *Candida dublinensis* (n=1, 3%). In total, three isolates of six *C. glabrata* species had dose-dependent sensitivity to fluconazole, and one *C. parapsilosis* was determined to be resistant.

**Conclusion:**

The COVID-19 patients who are admitted to ICU have many risk factors associated with candidemia. The most common risk factors for the development of candidemia were mechanical ventilation, diabetes mellitus, neutrophilia, and low hemoglobin level. The most frequently isolated species was *C. albicans*. Moreover, caspofungin was found to be the most effective drug *in vitro*. No significant resistance pattern was detected against the isolated species. It should be noted that risk-stratified antifungal prophylaxis in the ICU is possible.

## Introduction

The novel COVID-19 virus has affected our country since March 2020. It is currently responsible for the COVID-19 pandemic, which has resulted in a prolonged length of stay in a hospital or intensive care unit (ICU) [ 1 ]. The lung damage by the virus in ICU patients may be related to secondary infections after the start of the disease [ 2 , 3 ].

Candidemia is a frequent nosocomial bloodstream infection and is associated with high mortality for COVID-19 patients due to its increased incidence and early occurrence. 

The major risk factors for invasive candidemia are prolonged hospital stay, the use of broad-spectrum antibiotics, corticosteroids, immunosuppressive agents, and invasive procedures, such as intravascular catheters, mechanical ventilation, and dialysis [ 4 , 5 ].

According to the results of population-based studies, the incidence of candidemia has increased during the last decades. Recent studies have shown a higher incidence rate of candidemia in COVID-19 patients, compared to a historical cohort [ 6 - 8 ]. 

The main objective of this study was to examine the characteristics and the clinical features of COVID-19 patients with candidemia who were admitted to ICU in a tertiary care hospital to identify their species of *Candida*.

## Materials and Methods

### Patients and study design

This study was carried out in a tertiary referral hospital, with a total capacity of 1617 beds, located in the Central Anatolia region. 

A retrospective approach was undertaken which involved adult patients (>18 years) who were diagnosed with COVID-19 and hospitalized in ICU from July 2020 to January 2021.

Patients with candidemia were defined as the cases with the culture of a blood specimen that became positive for *Candida* species at least 48 hours after admission to the ICU. The control group comprised patients diagnosed with COVID-19 who did not have any infection or colonization with *Candida* spp. during their ICU stay. Candidemia was defined according to the standardized surveillance definitions of healthcare infection [ 9 ].

Patients who were not diagnosed with COVID-19 disease based on polymerase chain reaction (PCR) or clinical findings or were hospitalized at a different time in the same ICU or hospitalized in other ICU clinics, were excluded from the study. 

 The first episode of each patient with candidemia was included in the analysis. The data collection used for risk factor analysis continued until candidemia developed in case patients. On the other hand, for controls, the data collection continued for the total duration of their stay in the ICU.

### Data collection

The demographic characteristics, clinical findings, typical computed tomography (CT) findings, length of ICU stay, comorbidity, invasive procedures, and medication usage (antibiotics, antivirals, corticosteroıds, and tocilizumab) of the patients were obtained from the electronic hospital records.

All the cases were classified as having a severe/critical disease of COVID-19. Patients were considered to have severe illness if they had clinical signs of pneumonia (i.e., fever, cough, dyspnea, and fast breathing) as well as one of the following: respiratory rate > 30 breaths/min, severe respiratory distress, or SpO2 < 90% on room air. Critically ill patients were those who had acute respiratory distress syndrome (ARDS), septic shock, and/or multiple organ dysfunction [ 10 ]. The criteria for transfer to ICU included the need for invasive and noninvasive mechanic ventilation, administration of vasoactive agents, and development of shock.

The typical CT findings of COVID-19 were bilateral, subpleural, and peripheral ground-glass opacities and consolidation [ 11 ]. The recorded P/F ratio equals the arterial pO2 (“P”) from the arterial blood gas divided by the FIO2 (“F”) – the fraction (percent) of inspired oxygen that the patient is receiving expressed as a decimal (40% oxygen=FIO2 of 0.40). A P/F ratio of less than 300 indicates acute respiratory failure [ 12 ]. Acute Physiology and Chronic Health Evaluation (APACHE II) [ 13 ] and Sequential Organ Failure Assessment (SOFA) [ 14 ] scores were evaluated in the groups. 

Laboratory findings at the time of candidemia included complete blood count, liver enzymes, C-Reactive Protein, and procalcitonin. Neutropenia was defined as an absolute neutrophil count lower than 2000/mm^3^ [ 15 ] and lymphopenia was defined as a lymphocyte count lower than 1000/ mm^3^ in adults [ 16 ]. The COVID-19 was diagnosed based on positive real-time PCR (*Bioeksen*, Turkey) tests for SARS-CoV-2. Patients diagnosed with candidemia and *non-candidemia* were included in the study. All candidemia cases were defined as superinfection.

### Mycological identification

The blood specimens sent from ICU departments to the microbiology laboratory were incubated in the BacT/Alert 3D Automation System (Biomerieux, France). A positive signal obtained from the BACTEC automatic blood culture system was inoculated in Sabouraud Dextrose Agar (SDA, Oxoid, England) culture media with or without antibiotics from the bottles in which yeast cells had been detected by gram staining.

Fungal species identification has been performed by conventional and commercial methods.

The isolates were identified by the germ tube test, morphological images obtained from the Tween80 cornmeal agar, the capability of growth at 45 °C, urea hydrolysis, tolerance for 0.1% cycloheximide, as well as commercial methods, such as CHROM agar (Oxoid Brilliance™ *Candida* agar, England) *Candida* medium and *VITEK*® 2 (bioMérieux, France).

All isolates were cultured using SDA (Oxoid, Basingstoke, United Kingdom). These isolates were tested for susceptibility against amphotericin B (AMB), fluconazole (FLC), voriconazole (VRC), and caspofungin (CAS) by the E-test (Biomeriux, Marcy-l'Etoile, France) method. For the antifungal susceptibility testing, RPMI 1640 (Sigma Chemical Co., St Louis, Mo., USA) medium was prepared. Fort this purpose, 4 g L-glutamine, 34.5 g morpholinepropanesulfonic acid, 20 g glucose, and 17 g Bacto agar (Becton Dickinson and Company, Sparks, MD, USA) were dissolved in 1 L deionized water and autoclaved at 121˚ C for 15 min.

According to the manufacturer guidelines, the minimum inhibitory concentrations (MICs) were also determined by the E-test method. E-test strips of FLC (0.016-256 μg/ml), VRC (0.002-32 μg/ml), AMB (0.002-32 μg/ml), and CAS (0.002-32 μg/ml) were placed perpendicular to each other on an RPMI plate. In both tests, quality control was performed by the Clinical & Laboratory Standards Institute document M27-A3 using C. krusei ATTC 6258 and *C. parapsilosis* ATCC 22019 [ 17 ].

### Statistical Analysis

Statistical analysis was performed using IBM SPSS Statistics for Windows (Version 22.0). (IBM Corp. Armonk, NY: USA. Released 2013). The Shapiro Wilk test was used for the normality test of the parametric data. Numerical variables were specified as mean±SD and median (min, max). 

Comparisons between groups for data with a normal distribution were performed using Student’s t-test. The comparisons between groups for data that did not show a normal distribution were performed using the Mann-Whitney U test. It should be mentioned that a value of *p* ≤ 0.05 was considered statistically significant. Univariate and multiple binary logistics regression analyses and multiple regression analyses were performed to compare the variables.

### Ethical considerations

The Ethics Committee for Non-Invasive Clinical Research at the Kayseri City Hospital (2021-6/490) ethically approved this study.

## Results

In total, 126 COVID-19 cases were analyzed, including the candidemia group (mean age of 74.8±10, 61% male) and the non-candidemia group (mean age of 70.1±16, 55% male). In total, 44/126 (35%) of the patients were diagnosed with candidemia during the study period. Demographic information and comparisons of the study population are provided in Table 1. All the study populations had severe/critical COVID-19 illnesses. While the first group consisted of 44 candidemia patients with COVID-19 disease, the second group (control), consisted of 82 patients with COVID- 19 disease with non-candidemia. 

**Table 1:** Comparison of demographic and baseline characteristics of patients

Demographic characteristics	Candidemia n= 44	Non-candidemia n= 82	Total n=126	P
Mean age±STD (years)	74.8±10	70.1±15.9	71.7±14.0	0.07
18-65	5 (11)	12 (27)	27(21,4)	0.06
>65	39 (89)	60 (73)	99 (78,6)	0.06
Male gender	27 (61)	45 (55)	72 (57)	0.5
PCR positivity	42 (95,5)	82 (100)	124 (98,4)	0.1
Typical CT findings of COVID-19	35 (80)	54 (66)	89 (71)	0.1
Length of hospital stay, median days (range)	22 (2-76)	11 (1-56)	14 (1-76)	<0.01
ICU stay, median days (range)	15 (1-63)	8 (1-29)	10 (1-63)	<0.01
Mortality rate	32 (73)	73 (89)	105 (83)	0.02
APACHE (mean±STD)	15.5±7	18.9±7.6	17.7±7.5	0.1
SOFA (mean ±STD)	6±2.5	6.7±2.6	6.5±2.6	0.3
P/F ratio	58%	60%	59%	0.3
**Comorbidities**	39 (89)	62 (82)	101 (80)	0.1
Chronic Obstructive Lung Disease	11 (25)	24 (36)	35 (28)	0.2
Hypertension	27 (61)	30 (37 )	57 (45)	0.09
Diabetes mellitus	22 (50)	16 (19)	38 (30)	<0.01
Coronary artery diseases	11 (25)	15 (18)	26 (21)	0.4
Chronic renal failure	9 (21)	5 (6)	14 (11)	0.03
**Risk factors**				
Central venous Catheter	35 (80)	74 (90)	119 (94)	0.1
Total parenteral nutrition	39 (87)	71 (87)	110 (87)	1
Corticosteroıd Treatment*	44 (100)	71 (86)	115 (91)	0.01
Neutropenia (Neutrophils<2000/mm^3^ μL)	1 (2)	2 (2)	3 (2)	1
Lymphopenia (Lymphocytes<1000/mm^3^ μL)	31 (71)	54 (65)	85 (67)	0.6
Tocilizumab	8 (18)	15 (18)	23 (18)	0.9

PCR: Polymerase chain reaction, CT: computerized tomography, ICU: intensive care unit, APACHE: Acute Physiology and Chronic Health Evaluation, SOFA: sequential organ failure assessment

The candidemia group had 95.5% PCR positivity for all, and that rate was similar in both groups. A small number of the negative PCR patients had clinical and typical CT findings of COVID-19. At the same time, CT signs compatible with COVID-19 were similar between the two groups. The duration of hospital and ICU stay of patients with candidemia was significantly longer than the controls (11 days [1-56]). Candidemia was associated with an increased length of hospital stay (P<0.001). 

Diabetes mellitus (DM) 22 (50%) and chronic renal failure (CRF) 9 (21%) were more common in candidemia patients than in the other group (P<0.05). There were no significant differences between the groups in terms of the central vascular catheter, total parenteral nutrition, antibiotics, and antivirals (P>0.05). It should also be noted that systemic corticosteroids (Prednisone) usage was significantly higher in candidemia patients (P=0.01).

The median (min, max) total dosage of prednisone during the hospitalized days was 1120 mg (160-2240) higher in the candidemia group, compared to the other group with 480 mg (80-2000) (P<0.05). Tocilizumab usage and dosage were similar in both groups (total dosage was 213 mg in the candidemia group and 225 mg in the other group) (Table 2).

**Table 2:** Treatment of candidemia and other patients with COVID-19 in the intensive care unit

	Candidemia patients n=44 (%)	Other Patients n= 82 (%)	Total patients n=126 (%)	P
Antiviral agents*	44 (100)	82 (100)	126 (100)	-
Hydroxychloroquine	15 (35)	24 (30)	39 (31)	0.2
Favipiravir	42 (95)	81 (98)	123 (98)	0.1
Antibiotics**	44 (100)	82 (100)	126 (100)	-
Macrolides	38 (86)	69 (84)	107 (85)	0.1
Ceftriaxone	20 (45)	35 (42)	55 (44)	0.7
Fluoroquinolones	5 (11)	8 (9)	13 (10)	0.8
Piperacillin-Tazobactam	18 (41)	16 (19)	34 (27)	0.3
Carbapenems	32 (72)	60 (73)	92 (73)	0.1
Antifungal	23 (52)	-	23 (18)	
Fluconazole	2 (5)	-	2 (2)	-
Caspofungin	12 (10)	-	12 (10)	-
Voriconazole	1 (1)	-	1 (1)	-
Anidulafungin	7 (6)	-	7 (6)	-
L-Amphotericin B	1 (1)	-	1(1)	--
Mechanical ventilation	39 (89)	60 (73)	99 (79)	0,02

* Hydroxychloroquine 800 mg (loading dose) followed by 400 mg for five days; Favipiravir 1600 mg (loading doze) followed by 600 mg for five days.

** Clarithromycin 1 g/day, Ceftriaxone 2 gr/day, Piperacillin-Tazobactam 13.5 g/day, Carbapenems (Meropenem 3g/day, İmipenem-Silastatin 2 g/day, Fluoroquinolones (levofloxacin 500 mg/day, moxifloxacin 400 mg/day)

The laboratory characteristics and comparisons of the study population are provided in Table 3. The median white blood cell, neutrophile, and lactate dehydrogenase were higher in the candidemia group (P=0.02). However, the hemoglobin level was lower in the candidemia group (P<0.01). 

**Table 3:** Comparison of initial laboratory characteristics of Candidemia and others with COVID-19 in ICU

Laboratory findings Median (min, max)	Normal Range	Candidemia n=44 (%)	Other patients n=82 (%)	Total patients n=126 (%)	P
White blood cell count/μL	4500-10000	11600 (1280-64000)	8100 (470-33000)	8860 (470-64000)	0.02
Neutrophils/μL	1800-7500	9450 (680-45000)	6170 (80-29000)	7225 (80-45000)	0.02
Lymphocytes/μL	800-3200	570 (20-54000)	900 (10-4000)	790 (10-54000)	0.2
Hemoglobin (g/dL), mean±SD	13-17	10.9±2.3	12.1±2.1	12.2± 2.4	<0.01
Platelet count, x10^3^ μL	150-450x10^3^	155 (7-591)	180 (26-372)	170 (7-591)	0.3
Aspartate transferase (IU)	0-40	37 (8-843)	35 (15-678)	35(8-843)	0.2
Alanine aminotransferase (IU)	0-41	26 (5-1193)	23 (7-337)	23(5-1193)	0.07
Lactate dehydrogenase (U/L)	135-214	434 (45-2112)	347 (144-961)	377 (45-1112)	0.02
C-Reactive Protein (mg/dL)	0-5	89 (3-471)	78 (1.3-360)	83 (1.3-471)	0.4
Procalsitonin (µg/dL)	30-400	0.6 (0,04-63)	0.2 (0-87)	0.3 (0-87)	0.2

The need for mechanical ventilation therapy in candidemia patients (n=39, 89%) was higher, compared to the non-candidemia patients (n=60, 73%) (P=0.02). In the candidemia group, the 30-day mortality rate was

significantly higher than in the other group (73% vs. 89%, P=0.02). Moreover, it should be noted that the APACHE and SOFA mean scores and P/F ratio were similar between the two groups (P>0.05).

Univariate and multiple binary logistics regression analyses of diverse variables are shown in Table 4. Initially, in the univariate analysis, the variables were evaluated independently on an individual basis. Mechanical ventilation, diabetes mellitus, neutrophil count, and hemoglobin levels were associated with Candidemia according to the univariate and multiple analyses.

**Table 4:** Univariate binary logistics regression analysis of candidemia and others with COVID-19 in the intensive care unit

	Univariate		Multiple	
Variables	OR (95% CI)	*P*	OR (95% CI)	*P*
Length of hospital stay, median days (range)	1.053 (0.986-1.125)	0.3		
ICU stay, median days (range)	1.062 (0.954-1.182)	0.2		
Mechanical ventilation	15.2 (1.882-123.470)	0.005	0.27 (0.052-0.521)	<0.001
Diabetes mellitus	0.124 (0.022-0.699)	0.002	6.1 (1.76-10.7)	0.001
Chronic renal failure	1.047 (0.088-12.392)	0.2		
Corticosteroıd Treatment*	1,23 (0.9-1.238)	0.9		
Tocilizumab treatment	0.6 (0.173-2.789)	0.6		
Extended broad-spectrum antibiotics	8.9 (0.598-134.3)	0.1		
Median neutrophils (μL [min, max])	0.922 (0.425-2.002)	0.04	2.7 (1.02-6.35)	0.04
Hemoglobin (g/dL) mean±SD	0.642 (0.425-0.969)	0.01	6.1 (1.76-10.7)	0.001

* Administration of 0.5-1 mg/kg of prednisone equivalent in the last 30 days before candidemia, OR: odds ratio

**Figure 1:**
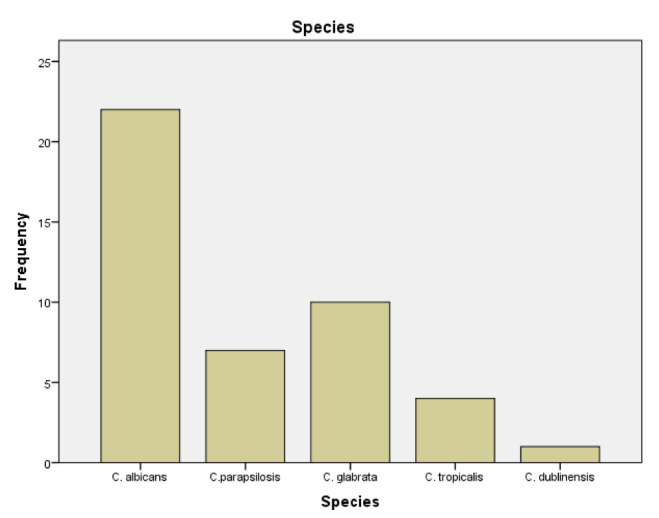
Candida species distribution in 44 candidemia patients

The incidence of candidemia (per 1000 admissions) was higher at 0.78 in 2021, compared to the pandemic period and 0.61 in 2019 before the pandemic period. In *total, 44 Candida* spp. were isolated from blood culture (Figure 1). The median time to the first isolation of yeast was 16 days (2-74 days). These strains were identified as follows: 22 (50%) *C. albicans*, 7 (16%) *C. parapsilosis*, 10 (23%) *C. glabrata*, 4 (9%) *C. tropicalis*, and 1 (2%) *C. dublinensis*.

The antifungal susceptibility tests for the 44 yeast isolates included in the study are summarized in Table 5 with the relevant MIC values. Antifungal resistance was not found against *C. albicans* or *C. tropicalis*, and low MIC levels were observed against all antifungal agents. Three isolates of the six isolated *C. glabrata* species had dose-dependent sensitivity to FLC, and one isolate, *C. parapsilosis*, was determined to be resistant.

**Table 5:** *In vitro* susceptibilities of the Candida isolates to four antifungal agents

		AMB (μg/ml)			CAS (μg/ml)			FLC (μg/ml)			VRC (μg/ml)	
Species (Number)	GM	MIC_50_	MIC_90_	GM	MIC_50_	MIC_90_	GM	MIC_50_	MIC_90_	GM	MIC_50_	MIC_90_
*Candida albicans* (40)	0.39	0.38	0.75	0.37	0.38	1	1.03	0.75	4	0.07	0.04	0.94
*Candida glabrata* (19)	1.25	1.5	4	0.31	0.5	0,75	12.4	16	32	0.3	0.38	1
*Candida parapsilosis* (26)	0.5	0.38	0.94	3.37	2	32	1.4	1.5	2	0.08	0.06	0.47
*Candida tropicalis* (7)	2.25	1	1.5	0.5	0.5	1	0.8	0.75	1	0.04	0.03	0.094

AMB; Amphotericin b, CAS; Caspofungin, FLC; Fluconazole, VRC; Voriconazole, GM; Geometric Mean, MIC; Minimum Inhibitory Concentration

For all isolates, no cross-resistance was encountered between FLC and VRC. None of the patients received any antifungal treatment in the ICU before the positivity of blood culture. In total, 23 patients out of all the candidemia groups had received antifungal therapy. Moreover, 19 patients were treated with an echinocandin, while four patients were treated with other antifungals. Since 11 patients died before the microbiological tests, they were not treated with antifungals.

## Discussion

This single-center study was conducted to determine the epidemiology and risk factors of nosocomial candidemia among COVID-19 patients. Demographic findings, laboratory values, and risk factors were reviewed for candidemia and non-candidemia in COVID-19 patients in the ICU. In our hospital, the incidence of candidemia in non-COVID-19 patients is 0.61 lower than the 0.78 found in COVID-19 patients. 

Other studies have also reported an increase in candidemia in patients with COVID-19, compared to non-COVID-19 patients [ 21 , 22 , 35 ]. The reason for the high incidence rate could be some risk factors from COVID-19. The potential risk factors for candidemia investigated in previous studies and prolonged hospital stays were identified as persistent risk factors.

Such patients are exposed to multiple risk factors for candidemia, such as a central vascular catheter, total parenteral nutrition, and antibiotics [ 18 , 19 ]. In this study, central vascular catheter, total parenteral nutrition, and antibiotics treatment did not differ between candidemia and non-candidemia groups, but the length of the stay was longer in the candidemia group.

Chronic conditions and other comorbidities have been reported in many cases of candidemia [ 20 , 21 ]. The relationship between DM and candidemia has been studied several times, especially since patients with DM are more sensitive to fungal infections than those without DM [ 22 , 23 ]. There are a few components in the pathogenesis of candidemia in patients with DM, especially *Candida* colonization, which is more common in patients with diabetes than in patients without DM. In this study, multiple underlying comorbidities were associated with candidemia; 39 (89%) cases had one or more comorbidities, compared to 62 (82%) controls (P=0.1). In this study, the number of those diagnosed with DM and CRF was significantly high (P<0.05) in candidemia patients.

Patients with COVID-19 often suffer from acute hypoxemic airway failure, followed by ARDS [ 24 ]. Mechanical ventilation is an essential tool in the management of respiratory failure in critically ill patients. Prolonged use of mechanical ventilation in patients increases the risk of colonization and hospital-acquired infection [ 25 ]. In this study, 79% of all the patients received mechanical ventilation for respiratory failure and mechanical ventilation was high in the candidemia group.

Tocilizumab is a monoclonal antibody against interleukin-6 receptor that can reduce macrophage activation syndrome-induced cytokine storm and is beneficial in some series of COVID-19 cases [ 26 ]. In animal studies, interleukin-6 deficiency has been reported to cause *Candida* infections [ 27 , 28 ]. 

In a study conducted during the pandemic, a high prevalence of candidemia was observed, concisely in patients treated with tocilizumab due to COVID-19. Based on the results of the aforementioned study, it can be speculated that the suppression of the IL-6 response might contribute to this blood infection [ 29 ]. Bishburg et al. [ 30 ] did not find a relationship between tocilizumab and candidemia infection in COVID-19 patients; this result is in line with those of the present study regarding the lack of difference between the groups. The reason for this may be the use of low-dose short-term tocilizumab in the patients.

Patients with severe COVID-19 can develop a systemic inflammatory response leading to lung injury and multisystem organ dysfunction. It was suggested that the high anti-inflammatory effects of corticosteroids could prevent or mitigate these destructive effects. One study found that all cases of candidemia occurred following high-dose corticosteroids used in the treatment of COVID-19 [ 31 ]. 

In other studies, no relationship was found between corticosteroid use and candidemia in COVID-19 patients [ 27 , 30 ]. In the present study, 100% of patients with candidemia and 86% of other patients used steroids and were identified as at risk for candidemia. Additionally, the total dosage of corticosteroids was higher in candidemia patients. The data variability of the studies may be due to the differences in the dose and time of corticosteroid administration determined by clinicians.

The present study found an approximately 30-day mortality rate in the control group which was higher than in the candidemia group. These differences are regarded as statistically significant (P<0.05). Bishburg et al. [ 30 ] revealed that the mortality rate of patients with COVID-19 was higher than that of patients with candidemia. They attributed the high fatality rate to extended hospital stays. Macauley et al. [ 32 ] found no significant difference in mortality rates between candidemia with and without COVID-19 disease.

The epidemiological patterns of *Candida* species are essential for selecting the appropriate antifungal agent during the pandemic process. Recently, countries have started to share their data through documentation. Only six candidemia cases were detected in one study in Iran and eight *Candida* isolates were identified in 1988 patients with COVID-19. They reported that the most often isolated species was *C. albicans* [ 33 ]. 

According to data from one tertiary hospital in the United States, 13 cases of candidemia with COVID-19 were detected [ 32 ]. They found that the most commonly isolated species were non-albicans *Candida*. However, according to data from Brazil and Italy, the most frequently isolated species were *C. albicans* [ 8 , 34 ]. In our study, 44 *Candida* isolates were identified in COVID-19 patients. Among these species, the most frequently isolated species was *C. albicans*, and the species that followed it were *C. parapsilosis*, *C. glabrata*, *C. tropicalis*, and *C. dublinensis* from highest to lowest, respectively. *C. parapsilosis* was the second most often-isolated species, especially in those, who used an intravascular device, in intensive care units [ 35 ]. Epidemiological data on candidemia in COVID-19 patients may differ between countries. This could not be fully explained, but it was considered that usual patient exposure, underlying diseases, and different hospital applications might have been the cause.

Antifungal resistance was not detected against *C. albicans* and *C. tropicalis* species. Three strains of *C. glabrata* were dose-dependent susceptible to FLC while one strain of. *C. parapsilosis* was resistant. All of these isolates were found to be sensitive to VRC. In our study, 23 patients with candidemia had used antifungals and the most commonly used antifungal was CAS, followed by anidulafungin. 11 patients were not treated with antifungals as they died before the microbiological tests.

We have not encountered a significant pattern of resistance to antifungals in blood-isolated Candida species in our hospital.

## Conclusion

We presented our experience with candidemia patients with COVID-9 in the intensive care unit at our hospital. The most common risk factors for developing candidemia were mechanical ventilation, diabetes mellitus, neutrophilia, and low hemoglobin according to the regression analyses. The most frequently isolated species was *C. albicans*. Multiple-drug resistance for the species was not found. The development of new research on the subject seems fundamental to detecting potential epidemiological changes. Local epidemiological information during the pandemic provides valuable information for the selection of empirical antifungal agents.

## Acknowledgments

This study was supported by Health Science University, Kayseri Faculty of Medicine (2021-6/490), Department of Infectious Diseases, Kayseri, Turkey.

## Authors’ contribution

Contributors ZBD and H. Sav were responsible for the organization and coordination of the trial. ZBD was responsible for the data analysis. H.Sipahioğlu, RCY and İ.Ç. developed the trial design. All authors contributed to the writing of the final manuscript. 

## Conflicts of interest

The authors declare that there are no conflicts of interest.

## Financial disclosure

No financial interests related to the material of this manuscript have been declared.
